# Drug-resistant HER2-positive breast cancer: Molecular mechanisms and overcoming strategies

**DOI:** 10.3389/fphar.2022.1012552

**Published:** 2022-09-23

**Authors:** Xiaofan Wu, Hongjian Yang, Xingfei Yu, Jiang-Jiang Qin

**Affiliations:** The Cancer Hospital of the University of Chinese Academy of Sciences (Zhejiang Cancer Hospital), Institute of Basic Medicine and Cancer (IBMC), Chinese Academy of Sciences, Hangzhou, China

**Keywords:** HER2, breast cancer, targeted therapy, drug resistance, overcoming strategies

## Abstract

Breast cancer is one of the most common malignancies and the leading cause of cancer-related death in women. HER2 overexpression is a factor for poor prognosis in breast cancer, and anti-HER2 therapy improves survival in these patients. A dual-targeted combination of pertuzumab and trastuzumab, alongside cytotoxic chemotherapy, constitutes the primary treatment option for individuals with early-stage, HER2-positive breast cancer. Antibody-drug conjugate (ADC) and tyrosine kinase inhibitors (TKI) also increase the prognosis for patients with metastatic breast cancer. However, resistance to targeted therapy eventually occurs. Therefore, it is critical to investigate how HER2-positive breast cancer is resistant to targeted therapy and to develop novel drugs or strategies to overcome the resistance simultaneously. This review aims to provide a comprehensive discussion of the HER2-targeted agents currently in clinical practice, the molecular mechanisms of resistance to these drugs, and the potential strategies for overcoming resistance.

## Introduction

Breast cancer is a diverse collection of tumors with various morphologies, biological makeups, and therapeutic approaches (Eroles et al., 2012). With more than 2.3 million new cases annually, in 2020, female breast cancer surpassed lung cancer as the most frequently encountered disease (Sung et al., 2021). Breast cancer is classified into five subtypes, including the luminal A subtype, luminal B subtype, basal-like subtype, human epidermal growth factor receptor 2 (HER2, also known as ERBB2 or CD340) overexpression subtype, and normal breast-like subtype, according to research carried out in the last few decades (Perou et al., 2000; Sorlie et al., 2001; Sorlie et al., 2003). HER2 plays a crucial role in cell growth, differentiation, motility, and invasion. It is one of the four transmembrane tyrosine kinase receptors (HER1-4) (Citri and Yarden, 2006). Approximately 25% of primary invasive breast tumors have HER2 overexpression or amplification, which are connected to aggressive disease and negative patient outcomes (Slamon et al., 1989). The first HER2-targeted medication authorized by the FDA is a monoclonal antibody called trastuzumab, which is humanized anti-HER2. Although trastuzumab significantly improves DFS(disease-free survival) in patients with HER2-positive breast cancer (Slamon et al., 2011), approximately one in four patients with the early-stage disease will relapse within the first decade after trastuzumab treatment. This indicates that resistance to treatment remains a challenge in HER2-positive breast cancer (Perez et al., 2014).

## The complex structure of HER2

**FIGURE 1:**
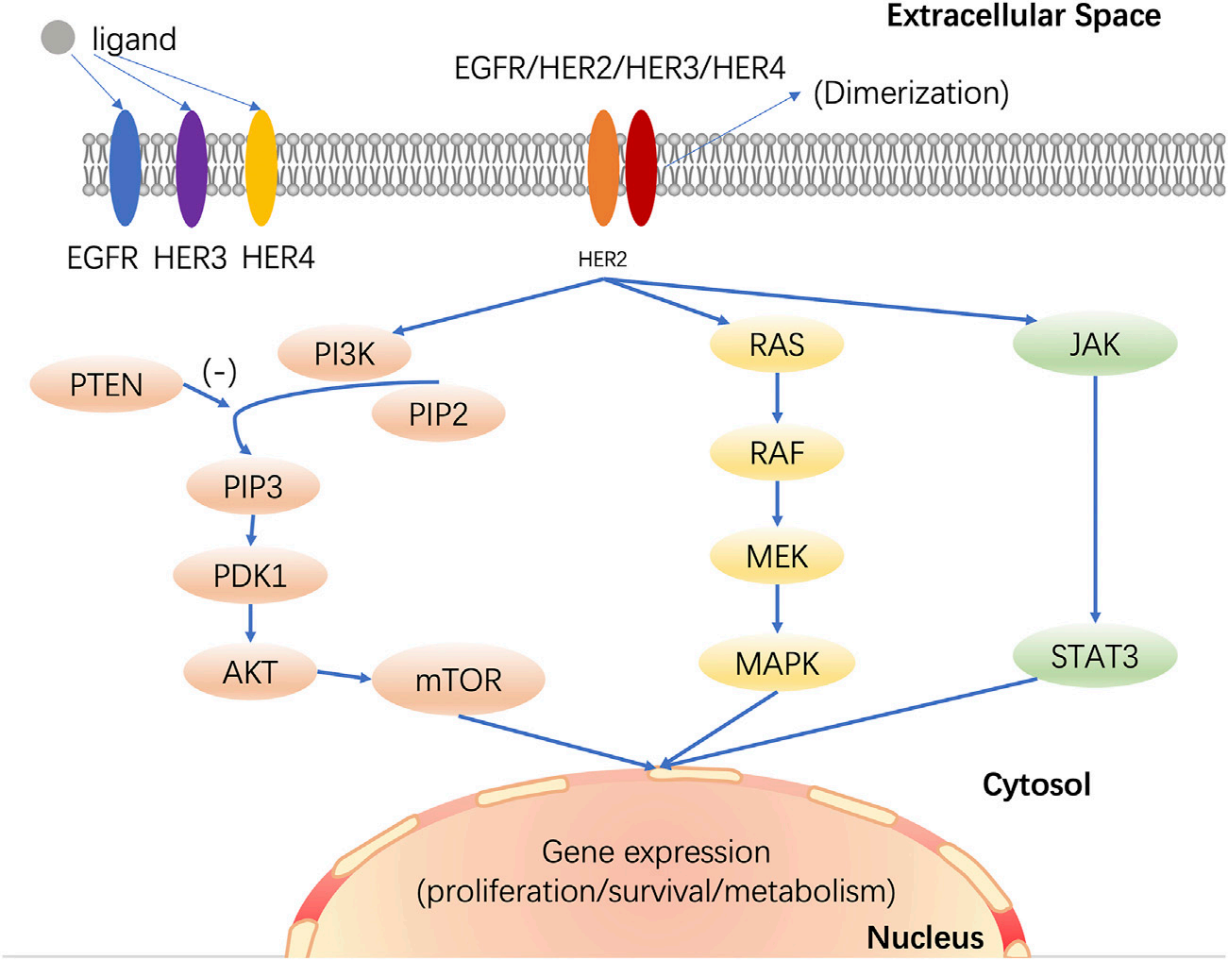
HER2 mediates multiple signaling pathways in breast cancer. HER2 dimerizes with other members of the EGFR family, activates downstream signaling pathways, and then regulates cell growth, proliferation, differentiation, apoptosis, and metastasis. PI3K: phosphoinositide 3-kinase; PTEN: phosphatase and tensin homolog deleted on chromosome ten; PIP2: phosphati-dylinositol-4,5-bisphosphate; PIP3: phosphatidylinositol-3,4,5-trisphosphate; PDK1: 3-phosphoinositide-dependent protein kinase 1; AKT: protein kinase B; mTOR: mammalian target of rapamycin; MAPK: mitogen-activated protein kinase; JAK: janus-activated kinase; STAT: signal transducer and activator of transcription.

HER2 typically is composed of an N-terminal extracellular domain, a transmembrane domain, an intracytoplasmic domain with tyrosine kinase activity, and a less conserved C-terminal tail (Carpenter, 1987). There are over eleven identified extracellular ligands in HER1, HER3, and HER4, but HER2 does not directly bind to any ligands. By homodimerizing or heterodimerizing with another type 1 receptor tyrosine kinases (RTKs), such as EGFR, HER3, or HER4, HER2 increases cell proliferation (Yarden and Sliwkowski, 2001). HER2-mediated activation of downstream signaling is ligand-dependent. In cancer, for example, ligand binding activates EGFR signaling, resulting in homodimerization or heterodimerization with another member of EGFR’s family and phosphorylation of EGFR, which activates downstream signaling, such as PI3K/AKT signaling cascades, RAS/RAF/MEK/MAPK signaling pathway, and JAK/STAT signaling pathway (Wallasch et al., 1995; Garrett et al., 2003). These signaling pathways regulate cell survival, proliferation, differentiation, motility, apoptosis, invasion, migration, adhesion, and angiogenesis (Figure 1).

## HER2-targeted therapies and mechanisms of resistance

FDA-approved HER2-targeted therapies include monoclonal antibodies (e.g., trastuzumab and pertuzumab), antibody-drug conjugates (e.g., T-DM1 and DS-8201), and small-molecule HER1/2 TKIs (e.g., lapatinib, neratinib, and tucatinib). In addition, multiple clinical trials of anti-HER2 targeted therapy are underway (Table 1). Patients with HER2-positive breast cancer now have much better prognoses thanks to the above therapies. However, resistance to these targeted therapies is frequently observed in clinical use.

**TABLE 1:** Clinical trials of anti-HER2 therapy.

Study name	Phase	Disease stage	Prior Anti-HER2 therapy	Treatment	Primary endpoint	Main result	References
NCT01772472	Phase III	Residual invasive disease after surgical	NO	T-DM1 (n = 743) vs. Trastuzumab (H) (n = 743)	3-years DFS	88.3% vs. 77.0%	Junttila et al. (2011)
NCT02568839	Phase II	Local	NO	Docetaxel + H + Pertuzumab (*p*) (n = 99) vs. T-DM1 (n = 98)	pCR	45.5% vs. 43.9%	Hatschek et al. (2021)
NCT01853748	Phase II	Local	NO	T-DM1 (n = 383) vs. Paclitaxel + *p* (n = 114)	iDFS	97.8% vs. 47%	Tolaney et al. (2021)
NCT01966471	Phase III	Early-stage	NO	Anthracycline + T-DM1 + *p* vs. Taxane + H + *p*	iDFS	No significant difference with iDFS	Krop et al. (2022)
NCT00374322	Phase III	Early-stage	NO	Lapatinib (L) vs. Placebo	DFS	No significant difference with DFS	Goss et al. (2013)
NCT01808573	Phase III	Metastatic	YES	Neratinib + Capecitabine vs. L + Capecitabine	PFS OS	8.8M vs. 6.6M 24.0M vs. 22.2M	Saura et al. (2020)
NCT00770809	Phase III	Local	NO	Taxane + H + L vs. Taxane + H vs. Taxane + L	pCR	57% vs. 45% vs. 30%	Fernandez-Martinez et al. (2020)
NCT02614794	RCT	Metastatic	YES	Tucatinib + H + Capecitabine vs. Placebo + H + Capecitabine	PFS	7.8M vs. 5.6M	Murthy et al. (2020)

### Monoclonal antibodies

Trastuzumab, as a recombinant human monoclonal antibody, binds to the extracellular domain IV of HER2 and inhibits the formation of homodimers, thereby blocking the generation of downstream signals (Cho et al., 2003). Pertuzumab binds to the extracellular domain II of HER2 and inhibits HER2 heterodimerization, thus inhibiting the generation of downstream signals activated by HER2 heterodimerization (Franklin et al., 2004). Additionally, trastuzumab and pertuzumab interact with immune effector cell Fc receptors to cause antibody-dependent cellular cytotoxicity (ADCC) (Lewis et al., 1993; Pietras et al., 1998; Gennari et al., 2004; Collins et al., 2012). The dual HER2-targeted combination of pertuzumab and trastuzumab, alongside cytotoxic chemotherapy, constitutes the first-line standard of neoadjuvant therapy for patients with HER2-positive breast cancer (Korde et al., 2021). However, dual blockade of pertuzumab and trastuzumab with docetaxel showed an 8-year overall survival rate of only 37% in HER2-positive metastatic breast cancer patients (Swain et al., 2020). The second-generation humanized monoclonal antibody margetuximab has been approved by the FDA for use in combination with chemotherapy for the treatment of previously treated metastatic HER2-positive breast cancer. Compared with trastuzumab, margetuximab increased binding to activating Fcγ receptor IIIA (CD16A) and decreased binding to inhibitory Fcγ receptor IIB (CD32B), which increased both natural and acquired anti-HER2 immune responses activation (Rugo et al., 2021).

Mechanisms of resistance to monoclonal antibodies are multi-factorial, including down-regulation or loss of HER2 expression, high expression of p95HER2, PTEN loss, and so on. P95HER2 is an NH2-terminally truncated form of HER2 that is highly tumorigenic but lacks the trastuzumab-binding ectodomain and the HER2 kinase domain. The downstream signaling of p95HER2 is constitutively activated by an intact intracellular kinase domain but is not responsive to trastuzumab (Scaltriti et al., 2010). The phosphatase and tensin homologous protein encoded by the tumor suppressor gene PTEN can activate the PI3K/AKT/mTOR pathway (Bermudez Brito et al., 2015). This antagonistic effect on PI3K can negatively regulate the PI3K/AKT/mTOR signaling pathway. Loss of PTEN leads to impairment of the pathway mentioned above and gives rise to malignancy, despite trastuzumab treatment (Ning et al., 2016). The S310F mutation in subdomain II of the extracellular domain of HER2 results in the substitution of serine at amino acid 310 with phenylalanine, which affects the binding of pertuzumab to HER2, resulting in pertuzumab resistance (Zhang et al., 2019). But this mutation did not affect the anti-HER2 effect of trastuzumab.

**FIGURE 2:**
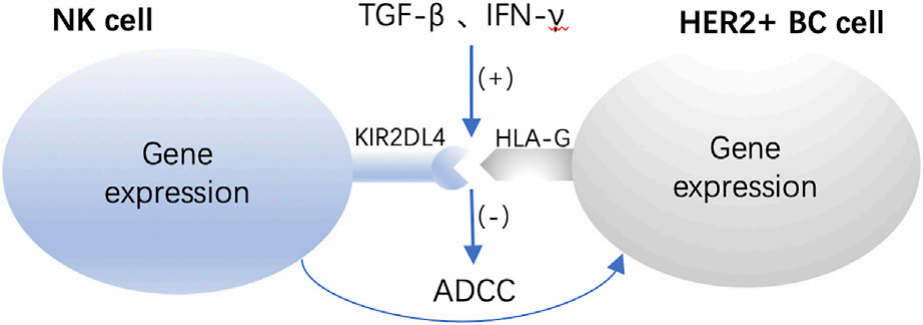
Enhanced HLA-G/KIR2DL4 signaling promotes resistance to trastuzumab. HLA-G disrupts ADCC by binding to the NK cell receptor KIR2DL4, making HER2-positive breast cancer cells resistant to trastuzumab.

Additionally, it has recently been demonstrated that human leukocyte antigen G (HLA-G) hinders ADCC by binding to the atypical KIR family receptor KIR2DL4 on NK cells, which may help explain why HER2-positive breast cancer is resistant to trastuzumab treatment (Zheng et al., 2021). KIR2DL4 is a member of the killer-cell immunoglobulin-like receptor family (KIR), and KIR2DL4 can activate ADCC of natural killer (NK) cells. Interferon gamma (IFN-γ) upregulates KIR2DL4 through JAK2/STAT1 signaling, and then KIR2DL4 cooperates with Fcγ receptors to increase IFN-γ secretion from NK cells, thus forming a feedback loop to promote ADCC (Zheng et al., 2022). Nonclassical histocompatibility antigen HLA-G is the only known ligand for KIR2DL4. HLA-G disrupts ADCC by binding to the NK cell receptor KIR2DL4, making HER2-positive breast cancer cells resistant to trastuzumab (Zheng et al., 2021). Not only that, but trastuzumab also induces excess TGF-β and IFN-γ production, enhancing HLA-G/KIR2DL4 signaling to promote this resistance mechanism (Figure 2). Therefore, the use of antibodies to deplete HLA-G or block the interaction of HLA-G and KIR2DL4 may be new approaches to overcome trastuzumab resistance.

### Antibody-drug conjugates

T-DM1 (Ado-trastuzumab emtansine), a conjugated antibody for the treatment of trastuzumab-resistant patients, combines trastuzumab with the cytotoxic medication DM1 (a microtubule polymerization inhibitor) and binds the two substances together covalently using a stable thioether linker (Girish et al., 2012). After binding to HER2, T-DM1 is internalized in cells, and the microtubule inhibitor payload DM1 can be cleaved from the antibody by lysosomal enzymes. Consequently, DM1 directly acts on cells that overexpress HER2 by inducing mitotic arrest and subsequent apoptosis (von Minckwitz et al., 2019). T-DM1 has been thoroughly investigated in breast cancer preclinical models (Junttila et al., 2011). In the phase III KATHERINE trial, patients with stage I to stage III HER2-positive breast cancer and residual invasive disease in the breast or axilla following neoadjuvant chemotherapy and HER2-targeted therapy were included; invasive DFS survival was significantly higher in the T-DM1 group than in the trastuzumab group, and the risk of distant recurrence was lower in those who received treatment (von Minckwitz et al., 2019). However, acquired T-DM1 resistance nearly always occurs in first responders (Krop et al., 2017). Since TDM1 is an ADC composed of trastuzumab and DM1, trastuzumab resistance and anti-microtubule drugs may contribute to TDM1 resistance. TDM1 must be internalized by cancer cells after binding to HER2 to have an anti-tumor effect, and this receptor-mediated endocytosis primarily uses the clathrin-mediated (CME) internalization pathway (Kalim et al., 2017). When T-DM1 is endocytosed, it is catabolized, resulting in the production of Lys-MCC-DM1 (active catabolite of T-DM1). However, lysosomal membranes are impermeable to these catabolites and require Lys-MCC to be transported through a transport mechanism, i.e., DM1 moves from the lysosome to the cytoplasm. The genetic weakening of the lysosomal membrane protein SLC46A3, a lysosomal transporter, inhibits the potency of T-DM1 (Hamblett et al., 2015). This may cause resistance to T-DM1. Caveolae is a highly hydrophobic membrane structure with a vesicular hollow of about 50–100 nm in diameter on the cell membrane, accounting for 20% of the cell membrane area. Caveolae is involved in the classical process of mediating endocytosis. Caveolin-1 (CAV-1) is a key protein molecule for caveolae biogenesis and function. CAV-1 is involved in the endocytosis of T-DM1 and is independent of clathrin-mediated endocytosis (CME) (Liang et al., 2021). Upregulation of CAV-1 enhances the sensitivity of HER2-positive breast cancer cells to T-DM1 by promoting endocytosis of trastuzumab (Chung et al., 2015). However, CAV1 knockout was insufficient to resensitize HER2-positive breast cancer cells to T-DM1 (Sung et al., 2018). This may be because another protein in the caveolae family compensates for the loss of CAV-1. Therefore, the expression level of CAV-1 may serve as an intrinsic biomarker for the sensitivity of HER2-positive breast cancer cells to T-DM1. Also, Caveolae may determine the sensitivity of cancer cells to specific types of ligation payloads from ADCs.

Trastuzumab deruxtecan (DS-8201) is a second-generation HER2-targeted ADC comprising a different payload and a cleavable linker that mediates the low-HER2-expressing cells to circumvent T-DM1 resistance mechanisms (Ogitani et al., 2016a). A portion of the payload (DXD) of DS-8201 can be released into the extracellular environment, affecting cells that do not overexpress HER2 (bystander effect) (Ogitani et al., 2016b). This enables DS-8201 to be active in breast cancer cells with low levels of HER2 or tumors resistant to T-DM1. In phase III DESTINY-Breast03 trial, in patients with trastuzumab and taxane-treated HER2-positive metastatic breast cancer, DS-8201 significantly reduced the probability of disease progression or death compared to T-DM1 (Cortes et al., 2022).

### Small-molecule tyrosine kinase inhibitors

Proteins called PTKs (Protein Tyrosine Kinases) catalyze the conversion of specific tyrosine residues in signal transduction molecules from their hydroxyl groups to the c-phosphate group of adenosine triphosphate (ATP). They transmit signals to regulate physiological and biochemical processes such as cell growth, differentiation, and death (Jiao et al., 2018). PTK disorders can lead to a range of diseases in the body. TKI refers to a series of oral small-molecule drugs that can promote cancer cell apoptosis and inhibit cancer cell proliferation. They have a homologous structure with ATP and can competitively bind to the intracellular ATP binding domain of EGFR, thus inhibiting tyrosine kinase phosphorylation and blocking downstream signaling (Yaish et al., 1988). It affects cancer cells with high HER2 expression by causing apoptosis and preventing their growth and migration.

Lapatinib is the first clinically-approved HER2-directed TKI. It is a dual reversible kinase inhibitor of EGFR and HER2 that can selectively inhibit HER2-amplified cells and function *in vitro* and *in vivo* with trastuzumab. Neratinib is an irreversible pan-HER TKI that covalently binds to specific cysteine residues (Cys-773 or Cys-805) in the ATP-binding pocket of RTKs and inhibits the tyrosine kinase activity of all HER family members. It was approved by the FDA in 2017 for expanded adjuvant therapy in patients with HER2-positive early breast cancer previously treated with trastuzumab (Deeks, 2017). Unlike other TKIs, tucatinib is a specific and reversible inhibitor of HER2 tyrosine kinase. It was approved by the FDA in 2020 in combination with trastuzumab and capecitabine for the treatment of adult patients with advanced, unresectable, or metastatic HER2-positive breast cancer (Lee, 2020).

In breast cancer, HER2 mutations occur in 3% of cases, most commonly in exon 19, with mutations in the L755 allele encoding the intracellular domain of tyrosine being the most common, accounting for 22% of mutations (Xu et al., 2017). The HER2 L755S mutation has been found to confer resistance to lapatinib in past *in vitro* mutagenesis screening studies. HER2 L755S is not only completely resistant to the dual regimen of laptinib + trastuzumab, but also to the regimen of T-DM1 combined with lapatinib. However, although L755S-mutated cell lines exhibited a lapatinib-resistant phenotype, the L755S mutation did not lead to cancer transformation (Bose et al., 2013). The HER2 L755 side chain is very close to the binding site of small-molecule kinase inhibitors, which affects the binding of TKI inhibitors to breast cancer cells. Not only that, the HER2-L755S mutation is an alternative driver event leading to TKI resistance through hyperactivation of the PI3K/AKT/mTOR and MAPK pathways, and this resistance can be overcome by combination therapy with related kinase inhibitors. This combined strategy warrants further preclinical or clinical studies to treat patients with the HER2 L755S mutation (Li et al., 2019). P27, a CDK inhibitory protein that belongs to the Cip/Kip family, is essential for regulating cell division, proliferation, and apoptosis. Lapatinib causes cell cycle arrest during the G1 phase, which prevents the proliferation of breast cancer cells that overexpress the HER2 gene, which is reliant on p27 upregulation. In addition to increasing p27 promoter activity and mRNA levels, lapatinib also causes p27 to become phosphorylated and prevents it from degradation (Tang et al., 2013). Poly (rC)-binding protein (PCBP1) belongs to the heterogeneous nuclear ribonucleoprotein (hnRNP) family, and downregulation of PCBP1 has been observed in a variety of cancers. PCBP1 binds to the p27mRNA 3′-UTR to stabilize p27 mRNA and increase p27 protein expression (Shi et al., 2018). Loss of PCBP1 expression first attenuates p27 expression at the post-transcriptional level, which may contribute to lapatinib resistance in breast cancer cells.

## Signaling pathways responsible for resistance to HER2-targeted therapies and potential overcoming strategies

### PI3K/AKT/mTOR

Evidence displays that PI3K/AKT/mTOR plays a fundamental role in various cellular activities, such as cell growth, cell proliferation, cell metabolism, apoptosis, and angiogenesis (Millis et al., 2016). PI3K is activated by specific receptors, which are triggered by extracellular ligands. PIP2 is phosphorylated by activated PI3K at position three of the inositol ring, creating PIP3, which recruits AKT and PDK1 to the plasma membrane via their pleckstrin homology-interacting domain (PH domain). AKT is phosphorylated by mTORC2 after it has been recruited to the cell membrane and activated AKT phosphorylates target proteins on the cell membrane, stimulating cell survival, growth, and proliferation (Fruman et al., 2017).

It has been found that the enhancement of the PI3K/AKT signaling pathway is one of the most common tumor sign alterations in breast cancer; more than 30% of invasive breast cancer involves PI3K/AKT mutation (Bachman et al., 2004). These alterations include functional mutations in PIK3CA, genes encoding the P110A catalytic subunit of PI3K, AKT1, or PIK3R1, as well as loss of PTEN, amplification of PIK3CA or AKT2, dephosphorylation of PIP3, and negative regulation of lipo-phosphatase by PI3K and so on (Nagata et al., 2004; Berns et al., 2007; Kataoka et al., 2010; Chandarlapaty et al., 2012; Majewski et al., 2015). HER2 transduces signals via the PI3K/AKT/mTOR pathway. Since trastuzumab inhibits PI3K from the upstream, any downstream mutations would make this upstream inhibition ineffective and result in drug resistance. Drug resistance to HER2-targeted therapy is linked to abnormal PI3K/AKT/mTOR or MAPK pathway activation which is downstream of HER2 (Fruman et al., 2017).

Recent preclinical studies have demonstrated that combination therapy with a PI3K inhibitor and trastuzumab is effective in HER2-positive breast cancer patients (Guo et al., 2021). However, adding the neoadjuvant PAN-PI3K inhibitor buparlisib to trastuzumab and paclitaxel in the NeoPHOEBE phase II clinical trial was not feasible to improve pathological complete response (pCR) in patients with HER2-positive primary breast cancer. In total, 19% of patients in this study discontinued the trial because of buparlisib toxicity. As the dose is limited by toxicity, this may reduce the efficacy of bupaxib (Loibl et al., 2017). Besides, studies using more specific second-generation PI3K inhibitors with better safety profiles (e.g., alpelisib, taselisib) are ongoing (Jhaveri et al., 2021; Perez-Fidalgo et al., 2022).

Everolimus, an mTOR inhibitor, is the first non-HER2-targeted therapy to address the underlying mechanisms of trastuzumab resistance. In the clinical trial BOLERO-1, adding everolimus to the combination of trastuzumab and paclitaxel provided a seven-month PFS (progression-free survival) benefit in patients with HR-negative, HER2-positive advanced breast cancer (Hurvitz et al., 2015). Adding the PAN-AKT inhibitor MK-2206 to standard neoadjuvant therapy also improved the estimated pCR rates in HR-negative and HER2-positive early breast cancer patients (Chien et al., 2020).

### IGF2/IGF-1R/IRS1

Insulin-like growth factor 2 (IGF2) is the endogenous ligand of IGR1-R. After IGF2 specifically binds to IGF1-R, it undergoes a conformational change and phosphorylates a specific intracellular signaling protein-insulin receptor substrate 1 (IRS1) and then triggers the downstream PI3K/AKT and MAPK signal pathways, thereby regulating cell growth, proliferation, invasion, and metastasis (Lero and Shaw, 2021; Zhang et al., 2022).

The proliferation and survival of HER2-positive breast cancer cells are inhibited by negative feedback inhibition of IGF2/IGF-1R/IRS1. Phosphorylated FOXO3a (forkhead box o3, a forkhead transcription factor from the FOXO subfamily) is elevated due to the transcriptional repression of PPP3CB (protein phosphatase three catalytic subunit beta, a subunit of serine/threonine protein phosphatase 2B) in trastuzumab-resistant cells. This causes the FOXO3a and miRNA negative feedback inhibitory loop to be broken; it also leads to the overexpression of IGF2 and IRS1 and the aberrant activation of the AKT/mTOR signaling pathway.

According to the above mechanism, targeting IGF2 will effectively overcome trastuzumab resistance. Xentuzumab is a humanized IgG1 monoclonal antibody against IGF1 and IGF2. This antibody inhibits IGF-1R and IR (insulin receptor) signaling by neutralizing the ligand. It has demonstrated effectiveness in numerous tumor models and is currently undergoing clinical trials (Luo et al., 2021). For the anti-HER2 and anti-IGF-1R combination therapy, cancer cells that are resistant to trastuzumab may be the best candidates (Sanabria-Figueroa et al., 2015). Another study found that dihydromyricetin can induce the expression of miR-98–5p and reduce the level of IGF2, thereby reversing the resistance of HER2-positive breast cancer cells to trastuzumab (Zhang et al., 2022).

### TGF-β/SMADs

The TGF-β family of proteins consists of TGF, TGF-β type I/II receptor, Smad family member 2 (Smad2), Smad3, and Smad4 and other relevant proteins (Massague, 1998). Serine/threonine kinases TβRI and TβRII form a hetero-tetrameric complex when TGF-β is presented to TβRII. Activated TβRII phosphorylates TβRI, which recruits, phosphorylates, and activates Smad2 and Smad3. Smad2 and/or Smad3 form a heterotrimer with Smad4. As the Smad complex accumulates in the nucleus, it drives transcription in concert with many specific transcription factors (Shi and Massague, 2003). When cells undergo the epithelial-mesenchymal transition (EMT), they lose their epithelial characteristics, such as cell polarity and specialized cell-cell adhesion, and instead exhibit migratory and invasive behaviors (Valcourt et al., 2005). The transforming growth factor TGF-β/Smad signaling pathway may be essential for EMT, according to mounting evidence (Islam et al., 2014). It has been determined that the functional interaction between HER2 and TGF-β signaling leads to breast tumor resistance to chemotherapy and HER2-targeted therapies (Wang, 2011). In trastuzumab-resistant cells, TGF-β/Smad3 signaling was significantly enhanced, and Smad3 phosphorylation levels were elevated. As is known, the miR-200 family suppresses tumor progression by regulating multiple genes associated with EMT, metastasis, and tumor angiogenesis. Indeed, miR-200c inhibits TGF-β signaling in HER2-positive breast cancer cells by inhibiting the translation of specific mRNAs, thereby increasing sensitivity to trastuzumab. Breast cancer cells’ development of trastuzumab resistance and aggressiveness may be caused by miR-200c′s defective expression (Bai et al., 2014).

## Role of tumor microenvironment (TME) in resistance to HER2-targeted therapies

**FIGURE 3:**
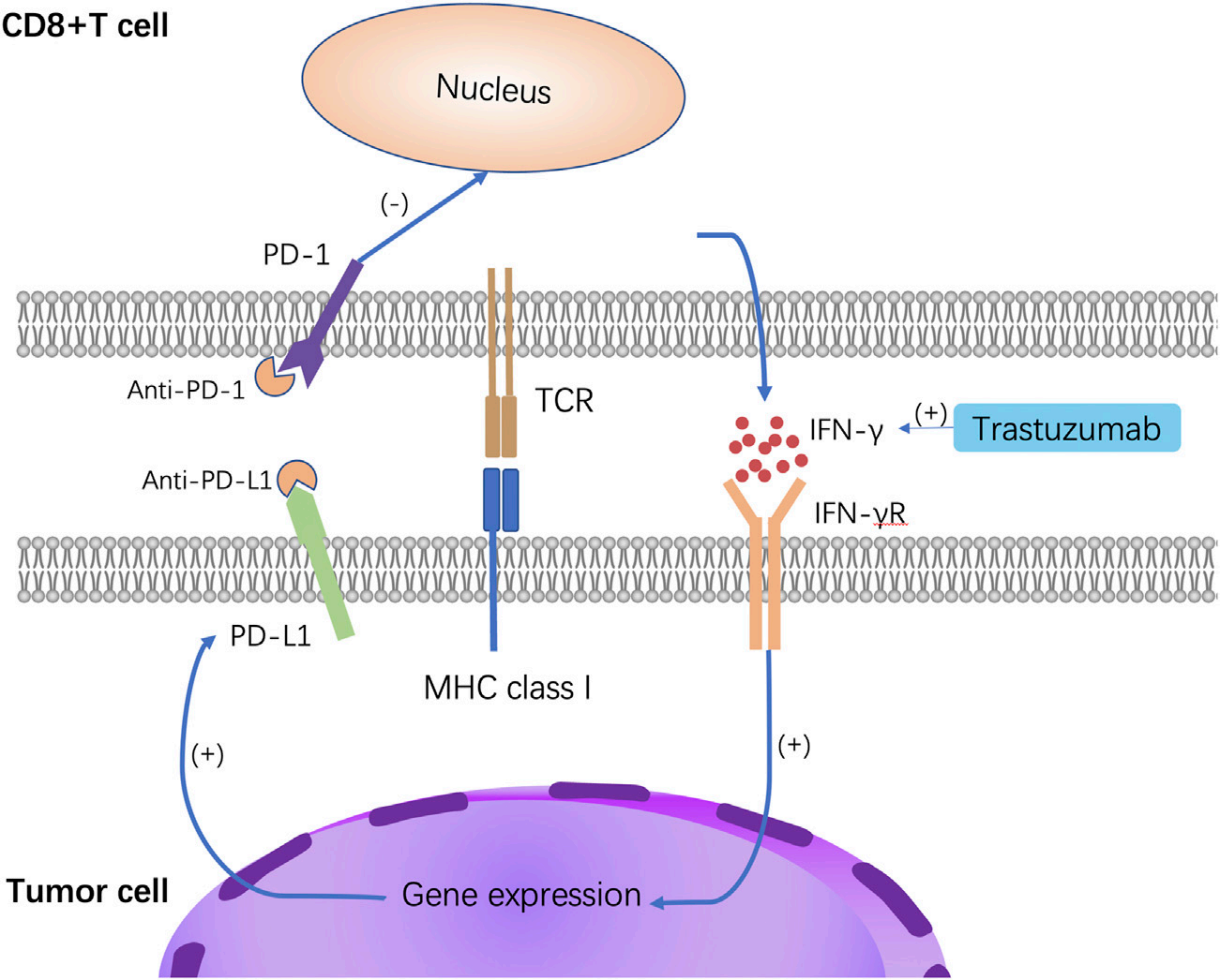
Mechanism of anti-PD-L1 therapy. After interferon exposure, tumor cells reactively express PD-L1, which binds to PD-1 on the surface of T cells and inhibits T cells. Anti-PD-1 or anti-PD-L1 antibodies can block the interaction of PD-1 with PD-L1 and reverse the inhibition of CD8^+^ T cells, thereby enhancing anti-tumor activity.

The tumor microenvironment (TME) includes cancer cells, immune cells, an extracellular matrix and interstitial tissue. The TME of HER2-positive breast cancer is complex, and the tumor-infiltrating lymphocytes (TILs) play the leading role in TME. Many lymphocytes are present within TILs, including CD4^+^ T cells, CD8^+^ T cells, CD20^+^ B cells, and CD68^+^ macrophages. In HER2-positive metastatic breast cancer, the higher the stromal TIL value, the higher the overall survival rate (Luen et al., 2017). Increasing the number of TILs and enhancing the ability of immune cells to kill tumors is key to overcoming drug resistance. Moreover, PD-1 is an essential immune checkpoint. After recognizing the MHC-I antigen, CD8^+^ T cells are activated to release IFN-γ and bind to the IFN-γ receptor, thereby inducing the expression of PD-L1 on tumor cells (Ribas, 2015). The combination of PD-1 and PD-L1 can induce cell apoptosis and inhibit the immune response in the immune microenvironment, and promote immune escape. By stimulating immune effector cells to release IFN, trastuzumab may increase the levels of PD-L1 in cancer cells that overexpress HER2, which could result in resistance to trastuzumab in anti-HER2 therapy (Chaganty et al., 2018). Anti-PD-1 or anti-PD-L1 antibodies can block the interaction of PD-1 with PD-L1 and reverse the inhibition of CD8^+^ T cells, thereby enhancing anti-tumor activity (Figure 3). The study by Mittal et al. demonstrated that anti-PD-L1 therapy could significantly improve anti-HER2 therapy (Mittal et al., 2019). Several phase I and phase II clinical trials of immunotherapy combined with anti-HER2 therapy are ongoing (Table 2), but some of the clinical trials yielded disappointing results, so further research is needed to screen the best benefit population of immunotherapy combined with anti-HER2 therapy.

**TABLE 2:** Clinical trials of immunotherapy combined with anti-HER2 therapy.

Study name	Stage	Treatment	References
NCT02129556	Phase Ib	Pembrolizumab + Trastuzumab	Loi et al. (2019)
NCT02924883	Phase II	Trastuzumab emtansine + atezolizumab vs. Trastuzumab emtansine + placebo	Emens et al. (2020)
NCT02649686	Phase Ib	Durvalumab + Trastuzumab	Chia et al. (2019)
NCT02605915	Phase Ib	Atezolizumab + (T-DM1 or Trautuzumab/Pertuzumab)	Hamilton et al. (2021)

## Conclusion and future directions

Although anti-HER2-targeted therapy has greatly increased survival for patients with advanced HER2-positive breast cancer, certain patients still exhibit clinical resistance to anti-HER2 treatments. The reasons for this resistance are manifold, such as up-regulation of HER2 expression, abnormal activation of HER2 downstream signaling pathways, deletion of tumor suppressor genes, cell cycle dysregulation, low infiltration of tumor immune cells, etc. Selective inhibitors of PI3K, inhibition of the IGF1R signaling pathway, and CDK inhibitors can sensitize HER2-positive breast cancer to anti-HER2 therapy, but these therapies have not yet been approved by the FDA and are still in clinical trials. Despite the potential for increased toxicity, the strategy of combining these drugs with HER2-targeted therapy may improve outcomes or even avoid chemotherapy in patients with HER2-positive breast cancer. As the comprehensive management of HER2-positive breast cancer has moved into the era of individualization and accuracy, research on the drug resistance mechanism of targeted therapy and drug combination therapy can provide new ideas for novel drug development and clinical use. We anticipate future studies that will help patients with HER2-positive breast cancer experience better prognoses.
